# Reply to: Sea-level rise may not uniformly accelerate cliff erosion rates

**DOI:** 10.1038/s41467-023-44150-w

**Published:** 2023-12-21

**Authors:** Jennifer R. Shadrick, Dylan H. Rood, Martin D. Hurst

**Affiliations:** 1 Department of Earth Science and Engineering, Imperial College London, London, UK; 2 School of Geographical and Earth Sciences, University of Glasgow, Glasgow, UK

**Keywords:** Climate change, Projection and prediction

**replying to** M. E. Dickson et al. *Nature Communications* 10.1038/s41467-023-44149-3 (2023)

We welcome the matters arising commentary by Dickson et al. in response to our paper “Sea-level rise will likely accelerate rock coast cliff retreat rates” published in Nature Communications in November 2022^1^. In their commentary, Dickson et al. highlight that the exploratory model that we used represents rock coast processes in a highly abstract manner. Further, they emphasise that the complex interaction of hydrodynamics, geology, and geomorphic processes is likely to mean that rock coast response to anthropogenic sea-level rise will not be consistent or uniform across highly varied environments. We agree with their observations, but emphasise that our results highlight the potential for rock coasts to be more sensitive to sea-level rise than previously identified, which motivates continued research endeavours to further our understanding of the complexities of rock coast erosion.

The exploratory model used in our study was developed by Matsumoto et al.^2^ and coupled with a cosmogenic radionuclide production model, in collaboration, by Hurst et al.^3^. It is the first process-based model used to interpret cosmogenic radionuclide concentrations at the coast and quantify long-term coastal cliff retreat rates. Optimisation of this process-based model has improved our understanding of rock coast behaviour by quantifying transient cliff retreat rates over millennial timescales using evidence from multiple empirical datasets^1,4,5^. Despite the advances made with the application of this model, Dickson et al. have highlighted the highly abstracted process representation required by the model, which is appropriate for exploratory modelling of rock coast behaviour, but suggest the model capability is limited at site-specific spatial scales and over decadal time scales. Indeed, we have used this model beyond its initial exploratory intentions by applying it to try and mirror the topography and cosmogenic radionuclide concentrations at specific coastal locations. The timescale over which cosmogenic radionuclides accumulate appreciable concentrations in a rock coast setting is centuries to millennia, and a process-based model of the topographic development of a cliff and shore platform needs to be highly abstract in order to be tractable over these timescales. The commentary by Dickson et al. highlights gaps in our ability to represent hydrodynamic and morphodynamic processes across the range of timescales needed to anticipate rock coast response to sea-level rise. As such, Dickson et al. have valid concerns that the model used, which was trained by measured cosmogenic radionuclide concentrations and observed topography, may not be suitable to allow us to forecast rock coast response to sea-level rise over the shorter decadal timescales during which we expect sea-level rise to accelerate in response to climate change. Nevertheless, alternative models that might be more suitable to simulating rock coast evolution over the shorter decadal timescales^6,7^ have not yet been coupled with predictions of cosmogenic radionuclide concentrations so could not be trained by our multi-objective approach that combines modern topography with a rich geochronological record of past erosion over timescales that allow for appreciable change.

Relative sea-level change around the UK has been relatively stable over the last 6-7 ka, which is the timeframe over which our model optimisations were focused. Future rates of SLR under the influence of anthropogenic climate change have not been experienced since the early Holocene, and as a result, our study sites do not provide a direct analogue for a rock coast that has experienced SLR during the Holocene at rates comparable to what is expected in the coming centuries. Thus, the comment made by Dickson et al. that “The model used by Shadrick et al., (2022) to predict future erosion rates was calibrated with observations from a time period with very different SLR rates than those forecast over the next century” is apt and accurate. SLR rates were decelerating in the period we used to calibrate the model, whereas SLR rates are accelerating in the future. Dickson et al. outline that there is complexity in the expected hydrodynamic and morphodynamic response to SLR that is not currently captured by any morphodynamic model of rock coast evolution. As the authors suggest, “we do not yet have a quantitative process-based description of the relationship between incident wave energy, sea level, and cliff erosion rates that is sufficiently detailed to inform robust future predictions of retreat rates”. Cliff retreat is episodic and retreat rates measured over short timescales (decades or less) are thus highly variable in time and space. There is a disconnect between the highly episodic, short-term cliff erosion events and the integrated long-term geomorphic development that we can learn about through using cosmogenic radionuclides under the external influence of climate change and sea-level rise.

We are grateful to Dickson et al. for highlighting that coastal response to future SLR will be variable across rock coasts and also controlled by factors other than the rate of sea-level rise as, for example, was highlighted in the discussion comparing our results and those of Swirad et al.^1,8^. Despite acknowledging the complexity of rock coast response to SLR, our key message in the paper is that there is evidence that cliff retreat rates at historically stable rock coasts will likely, but not exclusively, accelerate with future SLR. For example, we conclude that “Although there is evidence for controls, other than the rate of SLR on cliff retreat at other rock coast sites, these findings challenge conventional coastal management policies, in which rock coasts are considered stable environments compared to sandy coastlines. This study provides clear evidence that rock coasts should be included in future planning for climate change response and, importantly, we cannot use historical rates to assess the risk associated with rock coasts because climate change will transform the currently observed, stable behaviour of these globally ubiquitous coastlines”^1^. This conclusion of our study is significant since many rock coast settings are considered “almost-stable” environments^9^. Large stretches of the coast have exhibited next-to-no appreciable erosion during the timescale of historical observations^10,11^, yet this does not mean that there is no risk associated with cliff erosion, particularly under an uncertain and unprecedented future with anthropogenic climate-change-driven sea-level rise. Anticipating future rates of cliff retreat on eroding rock coasts requires knowledge of past erosion rates over ‘long’ (decades to millennia) timescales, long enough to integrate multiple erosion events.

In summary, we emphasise three key points relevant to the commentary of Dickson et al. First, process abstraction is a requirement for the modelling of rock coast evolution to be tractable at appropriate timescales for appreciable change. Second, there is a disconnect between the timescales of appreciable change on rock coasts (required to calibrate any models) and the scale of observations required to improve process representation in the model. And, finally, previous models of rock coast evolution have also suggested erosion is expected to increase with SLR, thus there is growing confidence in this concern. Thus, we feel it is important to change this narrative around rock coasts and improve awareness of this critical but understudied landscape. We hope this conversation will highlight important future research opportunities to help us better understand worldwide coastal cliff response to sea-level rise.

## Data Availability

The input data and calculations generated in this study are provided in the Supplementary Information of the original paper published in Nature Communications in November 2022^1^.

## Code availability

The coupled model by Hurst et al.^3^, model optimisation code by Shadrick et al.^4^ and documentation are publicly available online (10.5281/zenodo.5645478).
